# Geochemical signatures of sedimentary and diagenetic processes in the trace fossil *Rosselia* from the Pliocene in Taiwan

**DOI:** 10.1038/s41598-022-26772-0

**Published:** 2022-12-24

**Authors:** Yu‑Hsuan Liou, Ludvig Löwemark, Pei-Ling Wang, Shahin Dashtgard

**Affiliations:** 1 Paleoclimate Records in Shallow Marine Strata (PRISMS) Research Group, Department of Geosciences, National Taiwan University, No 1. Sec. 4 Roosevelt Road, P.O. Box 13‑318, Taipei, 106 Taiwan; 2 Paleoclimate Records in Shallow Marine Strata (PRISMS) Research Group, Earth Sciences, Simon Fraser University, Burnaby, BC V5A 1S6 Canada; 3 Paleoclimate Records in Shallow Marine Strata (PRISMS) Research Group, Institute of Oceanography, National Taiwan University, Taipei, Taiwan

**Keywords:** Carbon cycle, Palaeoceanography, Palaeoclimate, Biogeochemistry, Environmental sciences

## Abstract

Trace fossils are structures left in a substrate as the result of the activities of living organisms. The producer of the spindle-shaped trace fossil *Rosselia* incorporates fine-grained organic rich material into concentric layers surrounding the central shaft. Because *Rosselia* is common in stressed shallow marine environments where the preservation potential of organic material is generally poor, these trace fossils may act as natural archives, recording changes in the provenance of organic material. Carbon isotope values of organic carbon preserved in laminae of the studied *Rosselia* typically lie around − 26‰, suggesting a primary terrestrial source. However, increased levels of S and Ca detected from XRF scanning of the laminae indicate that at least some marine material is incorporated. Examination of a diagenetically altered specimen also demonstrates that both elemental composition and δ^13^C values can be substantially altered diagenesis. Nevertheless, the long stratigraphic range of *Rosselia*, from the Cambrian to the Present, and its ubiquitous occurrence in stressed shallow-marine settings make it a potentially powerful tool to reconstruct variations in the input of organic material in settings otherwise devoid of fine-grained organic matter.

## Introduction

**Figure 1:**
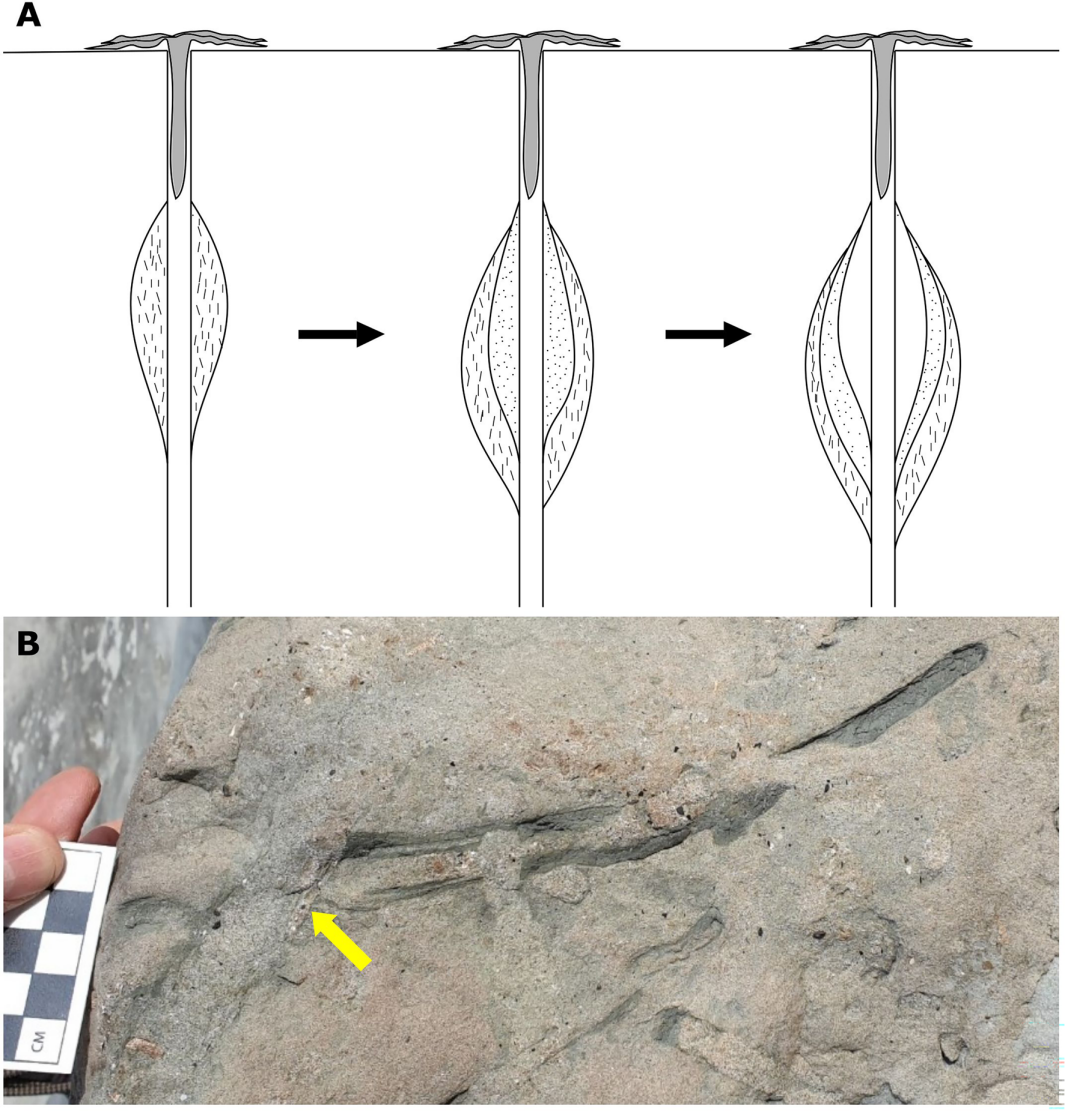
Field photo of *Rosselia* and conceptual illustration of its formation. (**A**) Cartoon illustrating how a marine bottom-feeder constructs a *Rosselia* burrow by ingesting detrital material at the sea floor and then deposits the resulting fecal material around the central shaft. In settings with rapid sediment deposition, this often leads to an upwards adjustment of the spindle (equilibrichnion) (adapted from^2^). (**B**) Photo of a vertical cross-section (top to the left, bottom to the right) of the lower part of *Rosselia,* illustrating the sand-filled shaft and distinct mud laminae. The top of the trace fossil is cross-cut by crustacean burrow (yellow arrow).

**Figure 2:**
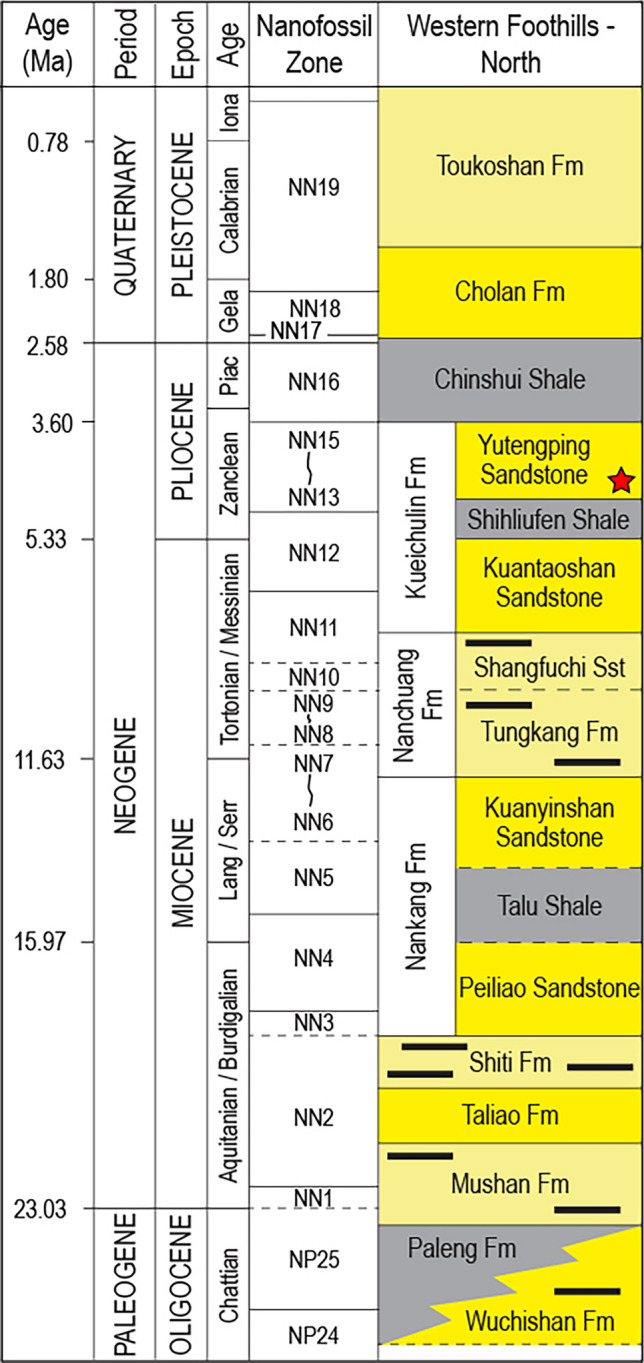
Stratigraphic chart for the studied region after^22^. Red star marks the stratigraphic position of the *Rosselia* samples analyzed in this study.

**Figure 3:**
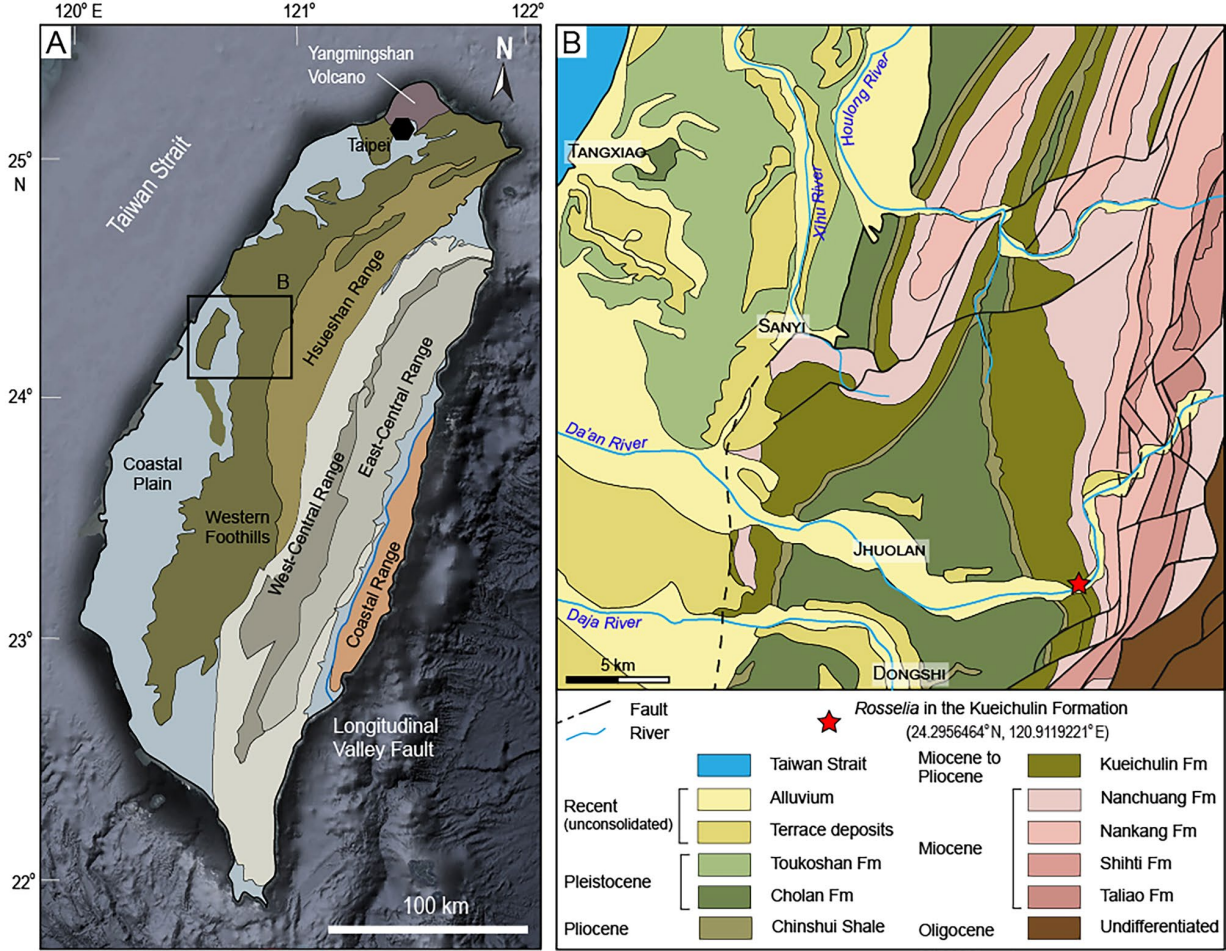
Geological maps of the study area based on^31,32^ (map drawn using https://www.adobe.com/adobe/illustrator). (**A**) Map showing the general geology of Taiwan. Black box indicates the region of panel B. (**B**) Geological map of the study region. Red star indicates sampling location of *Rosselia* analyzed in this study.

The trace fossil *Rosselia* Dahmer^1^ consists of a spindle shaped structure with a mostly vertical central shaft surrounded by concentric, fine-grained laminae composed of material collected on the sea floor by a detritus-feeding organism^2–4^ (Fig. 1). *Rosselia* is common in stressed shallow-marine environments such as delta fronts or storm-influenced shoreface deposits throughout most of the Phanerozoic e.g.,^5–9^. Because the laminae consist of fine-grained, organic-rich material, they also act as recorders of the organic sedimentary component in sandy environments where the preservation potential for organic matter is otherwise limited. Here we present high-resolution sediment geochemical parameters across two *Rosselia* specimens collected from the early Pliocene Kueichulin Formation (Fig. 2) in western Taiwan (Fig. 3). We use these data to test the hypothesis that the concentric layers act as short-term, high-resolution environmental recorders. We then assess how diagenetic alteration of the trace fossil influences the geochemical signals in the concentric layers. Finally, we discuss the value of *Rosselia* as paleo-environmental recorders through the Phanerozoic.

*Rosselia* occurs most commonly with trace-fossil assemblages typical of the *Skolithos* and *Cruziana* Ichnofacies. More recently, *Rosselia* became the namesake for the *Rosselia* Ichnofacies, which is considered to be typical of sandy, delta-front environments^9^. While the largest *Rosselia* may reach lengths up to one meter^2^ and diameters of more than 15 cm, typical sizes are 10–20 cm in length, and a 2–10 cm in diameter^1,2,10^. The concentric layers surrounding the central shaft comprise material that is considerably finer than the surrounding host substrate, and the central shaft is commonly filled with sediment similar in composition to the host substrate. Alternating colors of concentric laminations are the result of differences in grain size or sediment composition^2,11^.

*Rosselia* form through the repeated accretion of fine-grained material on the sides of the vertical shaft. As new material is added, older layers are pushed into the surrounding sediment resulting in the deposition of concentric laminae surrounding the shaft. The oldest laminae are furthest away from the center, and increasingly younger laminae occur towards the center of the spindle-shaped trace fossil (Fig. 1). The probable trace maker for *Rosselia* is a terebellid polychaete^2–4^, and the laminae in the burrow are believed to have formed as a result of the terebellid’s bottom-feeding habit: their tentacles were utilized to accrete sediment from the sea floor onto the inner wall of the burrow during the process of consumption. Modern terebellid worms often gather their food on the sea floor from the opening of their dwelling tube, resulting in a thickened tube wall at depth^12^. However, some polychaetes are known to convey large amounts of organic material collected on the sea floor into the sediment without ingestion^13^.

The fact that laminations in *Rosselia* are added sequentially over time suggests that they could act as high-resolution environmental recorders registering, for example, geochemical changes in the composition of fine-grained seafloor material collected by the trace-making organism. The concentric laminations of *Rosselia* would thus be similar to tree-ring records, but with the oldest layers furthest from the center of the burrow. The *Rosselia* tracemaker has been shown to survive severe erosional events caused by larger storms^14^, and individual animals lived for several years. Indeed, modern terebellid polychaetes have life spans that range from months to several years^15,16^. As well, *Rosselia* is a common trace fossil with a long stratigraphic range from Cambrian^17^ to the present^7,18^. Taken together, analysis of concentric laminations of *Rosselia* could provide snapshots as well as short, but highly resolved time series of variations in environmental conditions throughout the Phanerozoic. For example, in a paleogeographic setting such as the early Pliocene of proto-Taiwan, the steep topography of the rapidly uplifting orogen^19–21^ and heavy monsoon rains likely resulted in distinct seasonal alternations in the input of terrestrial vs. marine sourced organic carbon^22,23^. These seasonal swings would then be recorded as distinct shifts in the δ^13^C values of organic matter that make up the concentric layers.

Diagenetic processes may also influence and change the geochemical and isotopic signals recorded in the laminae. For example, isotopic studies on carbonates in the concentric layers of *Rosselia* revealed symmetrical variations in δ^18^O, indicating that the isotopic composition had been altered by a series of cementation events^11^.

## Results

### Morphological description

**Figure 4:**
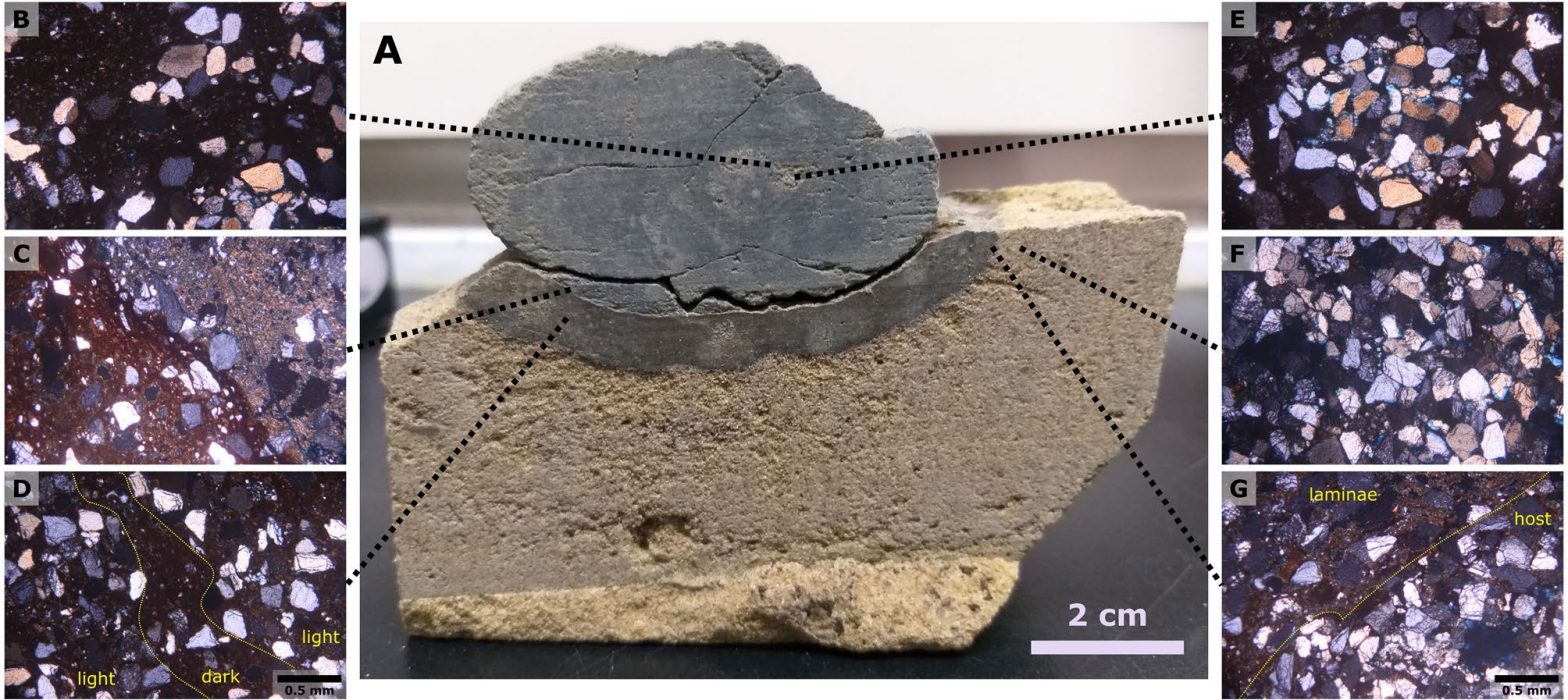
Polarized microscope images of mineral distributions and structures in thin sections derived from the Daan-4 specimen. Quartz is the dominant mineral of silt- and sand-sized material, with rare feldspar. The mineral grains are mostly angular and poorly sorted, and cemented by dark-colored matrix. (**A**) Cross-section view of the Daan-4 specimen. (**B**) Boundary between the central shaft and lamina, showing a high abundance of quartz grains in the central shaft (lower right corner), light-colored lamina (band of quartz grains stretching from left middle to upper right), and dark matrix-filled areas reflecting dark-colored laminae (upper left corner and a matrix-filled band between central burrow and light-colored lamina). (**C**) Boundary between unaltered (upper right corner) and diagenetically altered (lower left corner) laminae; the color of the mud matrices differ due to diagenetic alteration. (**D**) Diagenetically altered lamina showing the same pattern as unaltered laminae: high abundance of quartz grains reflect light-colored laminae, while matrix-filled bands reflect dark-colored laminae. (**E**) Central shaft and surrounding laminae; the central shaft is dominated by quartz grains. (**F**) Host rock (sandstone); dominated by densely packed quartz grains. (**G**) Boundary between diagenetically altered laminae and host rock.

Several specimens were collected from the Kueichulin Formation where it is exposed along the Da’an River in western Taiwan (see Figs. 2 and 3). Two *Rosselia* specimens are analyzed (Da’an-3 and Da’an-4), and both show an elliptical outline in horizontal cross-section, with the longest axis being about 70 mm in both cases. In both specimens, the concentric laminae are made up of alternating dark gray, greyish, and light grey layers that display a symmetrical distribution on both sides of the central shaft. Observed lamina thicknesses typically range from 0.5–1 mm, although individual lamina up to 6 mm were observed. These may, however, be the result of an amalgamation of several laminae. Thin-section analysis shows that while the central tube and surrounding host sediment are dominated by closely packed quartz grains, the dark colored concentric laminae are dominated by a dark matrix with only sparse quartz grains. In contrast, light colored concentric laminae contain high numbers of quartz grains, and greyish layers contain an intermediate number of grains (Fig. 4). This suggests that the color of the concentric laminae is controlled by the ratio of fine-grained matrix to coarse-grained particles. In Da’an-4, diagenetic alteration of the outmost ~ 1 cm of the specimen has resulted in a distinctly colored rim surrounding the entire trace fossil. Detailed thin-section analysis of this diagenetic rim show that its composition does not differ significantly from the inner, unaffected concentric layers.

### XRF core scanning

**Figure 5:**
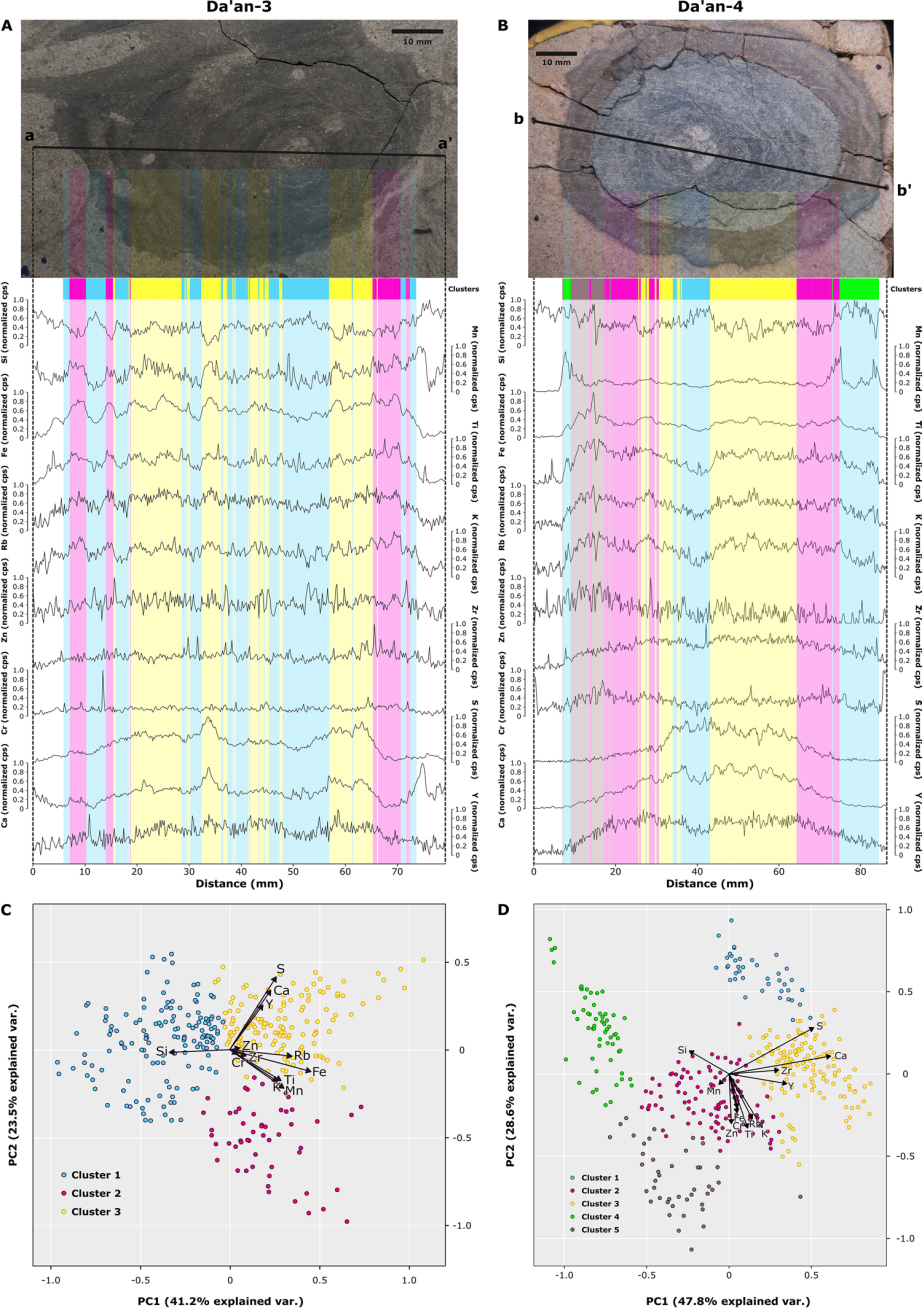
X-ray fluorescence data (normalized) of the Da’an-3 and Da’an-4 specimens derived using an Itrax core scanner. Principal component analysis (PCA) biplots of the XRF data of Da’an-3 and Da’an-4. Only elements with high reproducibility were selected as variables for PCA and cluster analysis (determined through duplicate scans), resulting in a total of 12 selected variables: Si, Mn, Fe, K, Rb, Ti, Cr, Zr, Zn, S, Ca, Y. Results of cluster analysis are presented in the figures through colors. In both *Rosselia* samples, the blue cluster overlaps with lighter laminae (including central burrow) and show high amounts of Si. Both the pink and yellow clusters overlap with darker laminae, showing high amounts of elements present in clay minerals (Ti, Rb, K).The yellow cluster is also rich in S and Ca. In Da’an-4, it is clear that the yellow cluster is darker in color than the pink cluster, and its green and beige clusters match the outer diagenetically altered laminae. The number of clusters was determined through the calculation of silhouette width, while cluster significance was verified through the multiple response permutation procedure (MRPP) and analysis of similarities (ANOSIM).

Elemental variations along transects through the two *Rosselia* specimens show a conspicuous correspondence between most elements and the concentric laminae. In particular, high concentrations of Si, typically related to mineral grains, correspond to the light- and greyish- laminae. Elevated concentrations in Ti, Rb, and K occur in the dark laminae, suggesting that clay minerals are an important component in these laminae (Fig. 5a, b). Multivariate analysis of elemental variations through the two trace fossils confirms this qualitative comparison. The elements group into distinct clusters corresponding to the central tube, the different concentric layers, and the surrounding host sediment. From the multivariate analysis, it is also clear that the light concentric laminae and central tube (blue cluster) are dominated by Si, and that darker laminae (yellow and pink clusters) are dominated by elements present in clay minerals (Ti, Rb, K). Moreover, the yellow clusters are specifically rich in S and Ca (Fig. 5c, d).

Both visual inspection of the elemental variations along the transect and the multivariate clusters show a generally symmetric distribution of darker and lighter laminae on both sides of the central shaft, although a number of discrepancies are evident. First, the outer, older laminae are more often discontinuous or difficult to trace around the trace fossil. Second, redox sensitive elements, such as Mn and Fe, display a conspicuous pattern in the outmost diagenetically influenced rim of the Da’an-4 specimen. Peaks at the edges of the diagenetically influenced laminae suggest that Mn, and to some extent Fe, have been mobilized from inside of the laminae and migrated towards the boundaries where it reprecipitated. This movement of cations resulted in strongly elevated Mn, and elevated Fe. For comparison, the Da’an-3 specimen lacks visual indications of diagenesis, and both Mn and Fe concentrations exhibit similar trends in concentrations to the other minerogenic elements.

### Total organic carbon and δ^13^C

**Figure 6:**
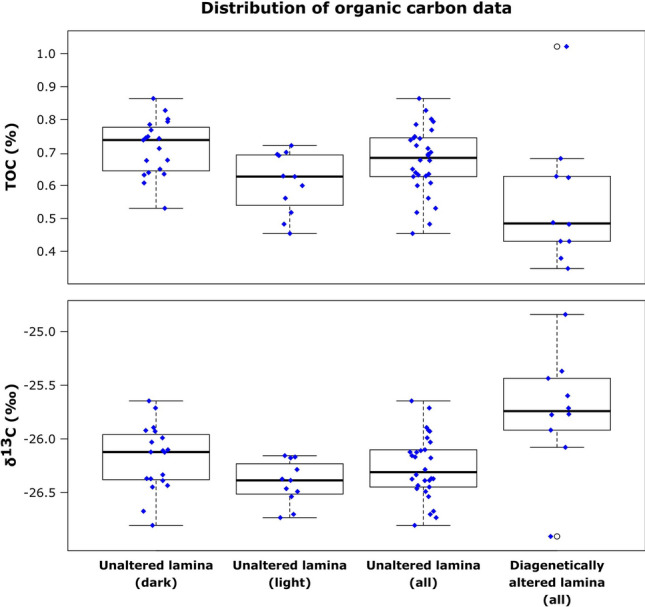
Boxplots of total organic carbon (TOC) values and stable carbon isotope ratios (δ^13^C) in dark-colored unaltered laminae, light-colored unaltered laminae, all unaltered laminae, and all diagenetically altered laminae of sample Da’an-4. Blue diamonds show values of all measured data points.

Total Organic Carbon (TOC) in both specimens shows considerably higher concentrations in darker laminae compared to lighter laminae, and both the host rock and central shaft contain even lower TOC. TOC content in dark laminae typically ranges from 0.5 to 0.9% (average 0.72%), and in lighter laminae TOC ranges from 0.4 to 0.75% (average 0.61%). Diagenetically overprinted laminae in Da’an-4 have TOC content that range from 0.3 to 0.7% (average 0.56%), although one outlier has a TOC > 1% (Fig. 6). Overall, the TOC contents of the diagenetically altered burrow margin in Da’an-4 is 0.1–0.2% lower than the light-colored and dark-colored unaffected laminae.

Similarly, there is a general tendency for lower δ^13^C values to occur in light-colored laminae (average − 26.4‰) compared to darker laminae (average − 26.2‰). Interestingly, the δ^13^C values of the diagenetically altered laminae in Da’an-4 (average − 25.7‰) are approximately half a ‰ higher than the δ^13^C values of the unaffected laminae (Fig. 6).

Statistical evaluation (Kruskal–Wallis Test) showed a statistically significant difference between dark and light laminae, as well as between unaltered and diagenetically influenced laminae for both TOC and δ^13^C (Supplementary data 1).

## Discussion

The general symmetry of the concentric laminae of the two studied *Rosselia* specimens observed both in thin sections and in elemental distribution measured using XRF core scanning suggest that each lamina represents one feeding season for the producer. Based on fair-weather deposits from an inner-shelf setting in the Western Gondwana Paraná Basin, southern Brazil, Netto et al.^5^ suggested that *Rosselia* is a stress-tolerant burrowing adaptation to environments often affected by erosional events in which the trace-making organism collects fine-grained material mobilized during storms (e.g., hyperpycnal flows). This interpretation is corroborated by the close association of *Rosselia* and sandy delta-front environments^9^. In fact, equilibrium response to erosional and depositional events by the *Rosselia* producers are known at least since the Ordovician^24^. Nara^25^ postulated that the enhanced influx of organic material during erosional events would provide beneficial conditions for the *Rosselia* trace-maker.

Consequently, *Rosselia* in the Kueichulin Formation in western Taiwan should preferentially record geochemical signals of material settling out after tropical cyclones. Unfortunately, despite the general symmetry of the laminae on both sides of the central shaft, it is rarely possible to systematically trace all laminae around the entire trace fossil. The paleoenvironmental signal recorded in the burrows, therefore, represents a series of snapshots rather than a continuous sequence.

Carbon isotope composition of the organic material in *Rosselia* are in the same range as those reported for the Kueichulin Formation by Dashtgard et al.^23^ who concluded that the organic material deposited in the early Pliocene proto-Taiwan Strait was almost entirely terrestrially sourced, and delivered primarily during tropical cyclones. The small shift (0.2‰) in δ^13^C values observed in the dark, fine-grained laminae (average − 26.2‰) vs light coarse-grained laminae (average − 26.4‰) could indicate a slight preference for the ingestion of marine-sourced organic-matter deposited in dark laminae. Presumably, the trace fossil producer had a preference for labile marine sourced carbon collected on the sea floor during quiet times over the more refractory carbon delivered by hyperpycnal flows from rivers cf.^26,27^. Additional support for an increased marine influence in the darker laminae comes from the distribution of the elements S and Ca. Both elements are largely of marine, biogenic origin cf.^28^, and show enhanced levels in the darker laminae, but considerably lower levels in lighter laminae and in surrounding host rock. Nevertheless, despite the small differences between light and dark laminae, the range of the isotopic composition recorded in *Rosselia* laminae suggest these structures provide information on shifting organic carbon sources in settings where the sandstones themselves contain very low TOC.

A limiting factor influencing the usefulness of δ^13^C trends derived from *Rosselia* is the diagenetic overprint. In specimen Da’an-4, the outmost approximately 1 cm of the trace fossil displays a diagenetically altered rim. Carbon isotope values from this rim consistently displayed approximately 1 ‰ higher δ^13^C values compared to values from inner, unaffected laminae. Consequently, care must be taken to not sample material for organic carbon isotope analysis that shows visible evidence of diagenetic alteration.

From the discussion above, the following conclusions regarding the use of *Rosselia* as a paleoenvironmental recorder are drawn:

- The concentric laminae in *Rosselia* are deposited in a semi-continuous way, providing a snapshot of environmental conditions on the sea floor.
- The dark, fine-grained laminae of *Rosselia* can be a source of information on paleo-sea floor values of δ^13^C in coarse-grained settings where TOC is otherwise too low for accurate measurements.
- Diagenetic processes may significantly alter both geochemical and isotopic signals recorded in the concentric laminae.

Because variations in the composition of sedimentary organic matter delivered to deltas and nearshore shallow marine environments reflect changes in the river catchment, organic matter can provide insights into processes such as provenance shifts due to orogenic processes, or paleoenvironmental changes caused by climatic shifts. Unfortunately, preservation of organic matter is usually poor in sandy environments. Here the preference of *Rosselia* for stressed, sandy environments cf.^9^, and the incorporation of substantial amounts of fine-grained, organic rich material provide a powerful tool to assess long term changes in the type and volume of organic matter delivered by ancient river systems. The long temporal range of *Rosselia* spanning most of the Phanerozoic^9,10^ allows this tool to be applied to a wide variety of scientific problems.

## Methods

Two samples of *Rosselia* preserved in fine-grained sandstone (Da’an-3) and coarse-grained sandstone (Da’an-4) host rocks, were taken from the early Pliocene Yutengping Sandstone Member of the Kueichulin Formation and analyzed in this study. Each sample was cut into several slabs perpendicular to the central shaft, exposing the concentric lamination of the spindle shaped part of the trace fossil. Each slab was then used for mineralogical and geochemical analyses.

Three analyses were applied to each sample: thin section analysis, X-ray Fluorescence (XRF) core scanning, and organic carbon analysis.

Thin sections of about 30 µm (μm) in thickness were made using Buehler’s PetroThin instrument, and focused on the central tube, the concentric laminae, and the host rock directly surrounding each *Rosselia* sample. Structural and mineralogical characteristics were then determined through observation of the thin sections in a polarized microscope.

The Itrax Core Scanner conducts XRF elemental analysis and produces semi-quantitative results in elemental content^29,30^. A slab from each *Rosselia* sample was scanned using the core scanner along straight lines that passed through the central burrow, perpendicular to the laminae, and into the host rock on either side. The Itrax has an analytical footprint of 0.2*4 mm and the samples were scanned with a step size of 0.2 mm. X-ray tube parameters include exposure time (5 s), voltage (30 kV) and current (50 mA). Duplicate scans were done to compare signal stability for each of the elements. The resulting elemental data was normalized before further statistical analyses were executed. Elements that proved unreliable due to the instrument design (Al, Ar, Mo, Ce, and W) and non-matching signal patterns between the duplicates were removed from the analysis. Observation points with uncommonly low overall signal strength and/or high Ar signal values most likely indicate cracks in the sample, were also removed. Cluster analysis and principal component analysis (PCA) were performed using the cleaned data to determine relative elemental fluctuation inside the *Rosselia* samples. The number of clusters was determined through calculating silhouette widths of each cluster and choosing the combination of highest values as the optimal outcome, while cluster significance was verified through the multiple response permutation procedure (MRPP) and analysis of similarities (ANOSIM).

The fluctuation of organic carbon in the laminae of each *Rosselia* sample was analyzed. Powdered samples were collected from each lamina by hand using a dentist drill, and samples were then soaked in 1 N HCl to remove carbonates. Total organic carbon (TOC) content was measured by an element analyzer (vario MICRO CUBE, elementar), and carbon isotope composition represented as δ^13^C value was analyzed by using an elemental analyzer (Flash EA, Thermo) connected with an isotope ratio mass spectrometry (Delta V, Thermo Finnigan).

## Supplementary Information

Supplementary Information.

## Data Availability

All data are available from the www.pangaea.de database.
